# PPAR-*γ* Ligand Inhibits Nasopharyngeal Carcinoma Cell Proliferation and Metastasis by Regulating E2F2

**DOI:** 10.1155/2019/8679271

**Published:** 2019-08-01

**Authors:** Ping-Li Yang, Jia-Shun Wang, Xiao-Mei Cheng, Jing-Cai Chen, Hui Zhu, Xiao-Lan Li, Li Cao, Wei Tang

**Affiliations:** ^1^Department of Otorhinolaryngology, The First Affiliated Hospital, Shihezi University School of Medicine, Shihezi, Xinjiang, China; ^2^Outpatient Department of Xinjiang Production and Construction Corp, Urumchi, Xinjiang, China

## Abstract

**Purpose:**

Peroxisome proliferator-activated receptor-*γ* (PPAR-*γ*) is a nuclear hormone receptor with a key role in lipid metabolism. Previous studies have identified various roles of PPAR-*γ* in cell cycle progression, cellular proliferation, and tumor progression. However, no report has described a role for PPAR-*γ* in human nasopharyngeal carcinoma (NPC). Notably, some studies have reported a relationship between PPAR-*γ* and E2F transcription factor 2 (E2F2), which has been identified as a regulator of cell cycle, apoptosis, and the DNA damage response. Notably, E2F2 has also been reported to correlate with a poor prognosis in patients with various malignancies.

**Methods:**

We used immunohistochemical (IHC) and western blot methods to evaluate PPAR-*γ* and E2F2 expression and function in nonkeratinizing NPC and nasopharyngitis (NPG) tissue samples, as well as western blotting and CCK8 analyses in the NPC cell lines, CNE1 and CNE2.

**Results:**

We observed lower levels of PPAR-*γ* expression in nonkeratinizing NPC tissues compared with NPG tissues and determined an association between a low level of PPAR-*γ* expression with a more advanced tumor stage. Furthermore, strong E2F2 expression was detected in nonkeratinizing NPC tissues. We further demonstrated that rosiglitazone, a PPAR-*γ* agonist, reduced E2F2 expression and proliferation in NPC cell lines.

**Conclusions:**

Our study results revealed a novel role for the PPAR-*γ*–E2F2 pathway in controlling NPC cell proliferation and metastasis.

## 1. Introduction

Nasopharyngeal carcinoma (NPC) arises from the epithelial and columnar cells of the nasopharyngeal mucosa. Histopathologically, the World Health Organization (WHO) has classified NPCs into three subtypes: type I, keratinizing squamous carcinoma; type II, nonkeratinizing carcinoma; and type III, basaloid squamous carcinoma. Although NPC is a rare disease worldwide, it has a high incidence in Southeast Asia, and type II is the most common form on both scales [1, 2]. Although NPC is a radiosensitive tumor, it is associated with relatively high rates of recurrence and distant metastasis at 2 years after radiotherapy. These factors underscore the need to identify factors related to the proliferation and metastasis of NPC, as well as potential therapeutic targets.

E2F transcription factor 2 (E2F2) has been identified as a regulator of cell proliferation, differentiation, and apoptosis [3]. Although many recent studies have associated E2F2 activity with inappropriate cell proliferation and/or apoptosis in various tumor types [4–6], the role of E2F2 in NPC remains unknown. Several research groups have identified E2F2 as a mediator in the ability of peroxisome proliferator-activated receptors (PPARs) *α*, *β*, and *γ* to regulate cell proliferation [7–9]. PPAR-*γ* is expressed strongly in adipose tissue and has been shown to play a key role in the onset of obesity [10], T2DM [11], metabolic syndrome [12], and cardiovascular disease [13], and the activated form plays an inhibitory role in cell growth and proliferation [14]. Increasing evidence suggests that PPAR-*γ* protects against tumors by inhibiting cell proliferation. However, the underlying mechanism remains unknown. Based on the abovementioned reports that PPAR-*γ* affects tumor proliferation and metastasis by targeting E2F2, in this study we investigated the expression patterns and activities of these proteins in NPC tissue samples and cell lines to determine the effects on tumor cell proliferation and differentiation.

## 2. Materials and Methods

### 2.1. Patient Tissue Samples

Fifty-two diagnosed nonkeratinizing NPC tissues and 34 diagnosed NPG tissues analyzed by the Department of Pathology of the First Affiliated Hospital of Shihezi University School of Medicine were collected from the Department of Otolaryngology of the same institution between April 2015 and January 2019. For each tissue sample, a portion was stored at −80°C prior to Western blotting and another portion was stored in formalin prior to immunohistochemistry (IHC) analysis.

### 2.2. Immunohistochemical Staining

Nonkeratinizing NPC and NPG tissues were fixed in 4% formalin for 12 h, dehydrated in a graded ethanol series (75%, 85%, 95%, and 100%), soaked in xylene, and embedded in paraffin. Subsequently, the samples were cut into 4 *μ*m sections and placed on glass slides, deparaffinized in xylene, and hydrated in a graded ethanol series (100%, 95%, 85%, and 75%). All slides were incubated in a citrate solution for heat-induced antigen retrieval and exposed to 3% H2O2 for 10 min to block endogenous peroxidases. After three washes with phosphate-buffered saline (PBS), the sections were incubated in a blocking solution containing bovine serum albumin (BSA) for 20 min, followed by exposure to primary antibodies against E2F2 (YT1443, Immunoway, rabbit polyclonal, 1:200 dilution) and PPAR-*γ* (Novusbio, rabbit polyclonal, 1:100 dilution) at 4°C overnight. The antigenic sites were visualized using a diaminobenzidine (DAB) kit. The nuclei were counterstained with hematoxylin. Finally, the slides were dehydrated in a graded ethanol series and xylene and observed under a microscope. The expression levels of E2F2 and PPAR-*γ* in the tissues were classified as follows: IOD SUM/Area SUM <0.02, low expression, or IOD SUM /Area SUM ≥0.02, high expression.

### 2.3. Real-Time RT-PCR

Total RNA was isolated from the NPC and NPG tissues, which was prepared using an E.Z.N.A. total RNA kit I (Omega Bio-Tek). The harvested tissue was homogenized in TRK lysis buffer, followed by RNA isolation and purification. A TaKaRa LA Taq® reverse transcription kit was applied to reverse transcribe mRNA from NPC and NPG tissues into cDNA. After that, quantitative real-time-PCR was performed. The primer sequences for human GAPDH, E2F2, and PPAR-*γ* used in this study were GAPDH forward: 5′-TCAAGAAGGTGGTGAAGCAGG-3′; GAPDH reverse: 5′-TCAAGGTGGAGGAGTGGGT; E2F2 forward: 5′-CTGAAGGAGCTGATGAACACG-3′; E2F2 reverse: 5′-CCCTTGGGTGCTCTTGAGATA-3′; 15-PGDH forward: 5′-GCATAGTTGGATTCACACGCT-3′; 15-PGDH reverse: 5′-TTGGCAATCAATGGTGGGTC-3′. Relative expression level was calculated for each gene by the 2^−ΔΔCT^ method with GAPDH for normalization.

### 2.4. Cell Lines and Cell Culture

The human NPC cell lines CNE1 (well differentiated) and CNE2 (poorly differentiated), which can represent the characteristics of NPC cell lines with different degree of differentiation, were cultured in Dulbecco's modified Eagle's medium (DMEM) medium (Hyclone, USA) supplemented with 10% fetal bovine serum (Gibco, USA) and 1% penicillin/streptomycin (Hyclone, USA). All cells were cultured in an atmosphere of 5% CO2 at 37°C. NPC cell lines, CNE1 and CNE2, were obtained from the cancer center of Union Hospital (Wuhan, China).

### 2.5. Western Blotting

Nonkeratinizing NPC and NPG samples and CNE1 and CNE2 cells incubated with or without PPAR-*γ* ligand were collected and lysed in radioimmunoprecipitation assay (RIPA) buffer (Beyotime, China) containing a phosphatase inhibitor and phenylmethanesulfonyl fluoride. The protein amounts in the lysates were quantified using a BCA protein assay kit (Beyotime, Haimen, Jiangsu, China), and 20–50 *μ*g of total proteins per sample were loaded onto 10% sodium dodecyl sulfate-polyacrylamide gels and transferred to polyvinylidene difluoride membranes. Subsequently, the membranes were blocked in a 5% solution of nonfat milk powder at room temperature for 1 h and subsequently incubated overnight at 4°C in dilutions of the following primary antibodies: E2F2 (YT1443, rabbit polyclonal, 1:1000 dilution), PPAR-*γ* (NR1C3, rabbit polyclonal, 1:500 dilution), and GAPDH (KM9002, mouse monoclonal, 1:4000 dilution). Next, the membranes were washed and incubated with species-specific, horseradish peroxidase-conjugated secondary antibodies for 1 h at room temperature. The proteins were finally visualized using the Enhanced Chemiluminescent Plus reagent (Beyotime, China). The target protein levels in each sample were quantified densitometrically and normalized against GAPDH levels in the same sample.

### 2.6. Cell Counting Kit-8 Proliferation Assay

CNE1 and CNE2 cells were seeded in 96-well plates at a density of 10^3^ cells/well and incubated overnight. Next, 5, 10, and 20*μ*mol rosiglitazone (Rog), a PPAR-*γ* agonist, and/or 100 nmol GW9662, a PPAR-*γ* antagonist, were added to the wells. At 24, 48, 72, and 96 h, indicated wells were washed with PBS, and the medium was replaced with 100 *μ*L of fresh medium plus 10 *μ*L of CCK-8 kit reagent. The cells were further cultured for 4 h, and the optical density (OD) of each well was measured at 450 nm using a SynergyMx MultiMode Microplate Reader (Biotek, USA) at 0, 0.5, 1, and 2 h. OD values of different density Rog groups against control group were analyzed

### 2.7. Flow Cytometric Analysis

The quantification of apoptotic cells was performed with the rh Annexin V-FITC Detection Kit (Antgene) according to the manufacturer's instructions. CNE1 and CNE2 cell lines were harvested by trypsinization and washed twice in cold PBS. Staining was performed with rh Annexin V-FITC and propidium iodide. Apoptosis was determined by calculating the percentage of apoptotic cells relative to the total number of cells. The results were confirmed in at least three independent experiments.

### 2.8. Statistical Analyses

All statistical analyses were performed using SPSS (version 17.0; IBM Corp, USA). The chi-square test was used to compare the levels of expression of E2F2 and PPAR-*γ* in nonkeratinizing NPC and NPG samples and the respective correlations with the clinicopathological features of NPC patients. The unpaired Student's t-test was used to compare E2F2 and PPAR-*γ* expression between groups of CNE1 and CNE2 cells. For all analyses, a P value of <0.05 was considered to indicate a statistically significant difference.

## 3. Results

### 3.1. Expression of E2F2 and PPAR-*γ* in Undifferentiated NPC and NPG Tissues and Correlations with the Clinicopathological Features of NPC Patients

IHC was used to evaluate the expression of E2F2 and PPAR-*γ* proteins in 52 nonkeratinizing NPC and 34 NPG tissue samples. Strong E2F2 expression (IOD/Area ≥0.2) was observed in 63.46% (33/52) of nonkeratinizing NPC tissues, compared with 17.65% (6/34) of NPG tissues (P <0.01). Strong PPAR-*γ* expression (IOD/Area ≥0.2) was also observed in 28.85% (15/52) of nonkeratinizing NPC tissues, compared with 76.47% (26/34) of NPG tissues (P <0.01) (Table 1). Both differences were significant.

We further analyzed the associations of E2F2 and PPAR-*γ* expression with the clinical parameters in patients with nonkeratinizing NPC. Although no correlations were observed with sex, age, or T (tumor) classification, we observed a positive correlation between E2F2 and nonkeratinizing NPC staging, namely the N (lymph node metastasis) (N0–N1, 22/40(55.00%) vs. N2–N3, 11/12(91.67%); P <0.05) and M (distant metastasis) classifications (M0, 23/52(54.76%) vs. M1, 10/10(100%); P <0.05). PPAR-*γ* expression was only found to correlate positively with the M classification (M0, 15/42 (35.71%) vs. M1, 0/10(0); P <0.05). These results strongly indicate that E2F2 contributes to the proliferation and metastasis of nonkeratinizing NPC and is associated with the disease stage.

### 3.2. Immunohistochemical Staining of E2F2 and PPAR-*γ* in Nonkeratinizing NPC and NPG Tissues

**Figure 1:**
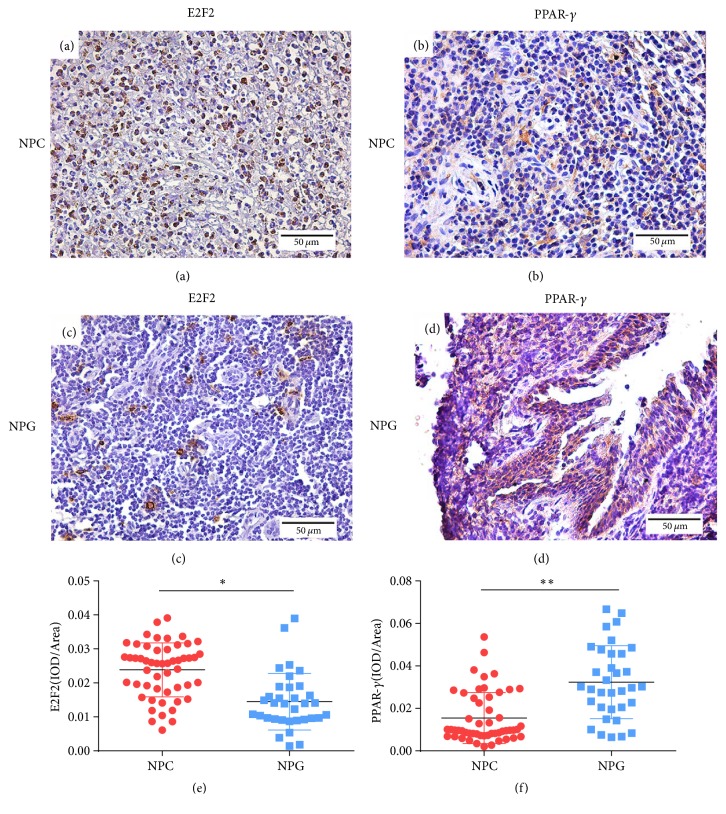
*E2F2 and PPAR-γ protein expression in nonkeratinizing nasopharyngeal carcinoma (NPC) and nasopharyngitis (NPG) tissues*. Immunohistochemistry was performed to detect E2F2 and PPAR-*γ* protein expression in nonkeratinizing NPC tissues (a, b) and NPG tissues (c, d). Scale bar, 50 *μ*m (e, f). The immunohistochemistry data were analyzed semiquantitatively to determine the E2F2 and PPAR-*γ* expression levels in nonkeratinizing NPC and NPG tissues. Data are shown as means ± standard deviations. *∗∗*P <0.01.

IHC was used to evaluate the expression and localization of E2F2 and PPAR-*γ* in tissue samples. Notably, we observed strong E2F2 expression in nonkeratinizing NPC tissues (Figure 1(a)), compared with NPG tissues (Figure 1(c)). Although E2F2 localized in both the nuclei and cytoplasm of the former tissues, it was most strongly expressed in the nuclei. We further observed a trend toward decreased PPAR-*γ* expression in nonkeratinizing NPC tissues (Figure 1(b)), compared with NPG tissues (Figure 1(d)). A semiquantitative evaluation revealed significantly stronger E2F2 expression in nonkeratinizing NPC tissues, compared to NPG tissues (Figure 1(e)) (P <0.01), as well as a significant difference in PPAR-*γ* expression between these tissue types (Figure 1(f)) (P <0.01).

### 3.3. Expression of E2F2 and PPAR-*γ* in Nonkeratinizing NPC and NPG Tissues

**Figure 2:**
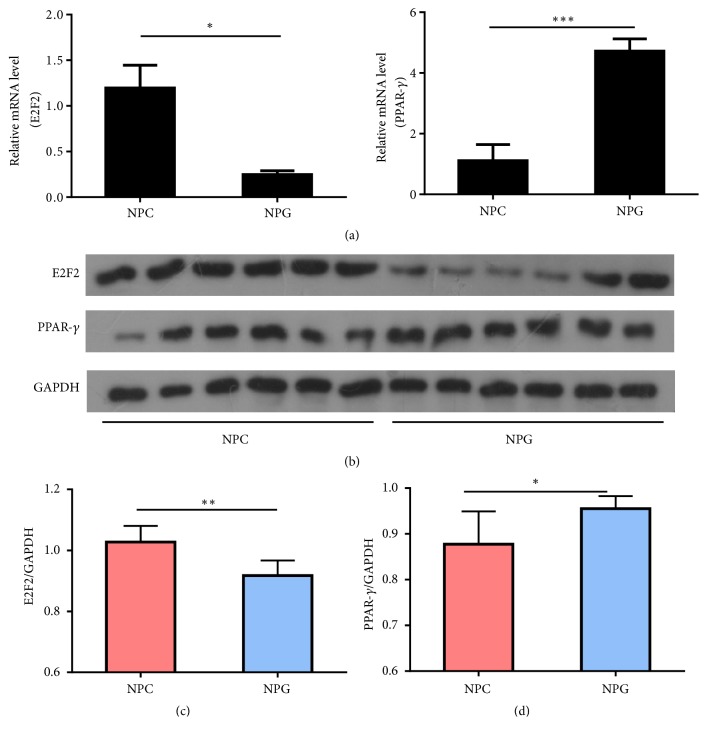
*E2F2 and PPAR-γ protein expression in nonkeratinizing nasopharyngeal carcinoma (NPC) and nasopharyngitis (NPG) tissue lysates*. (a, b) RT-PCR and Western blotting were performed to detect the expression of E2F2, PPAR-*γ*, and GAPDH in lysates of nonkeratinizing NPC and NPG tissues. (c, d) Quantitative analyses of the E2F2 and PPAR-*γ* expression levels in nonkeratinizing NPC and NPG tissues. Data are expressed as means ± standard deviations. *∗*P <0.05; *∗∗*P <0.01.

The mRNA expression of E2F2 in NPC is significantly higher than NPG, while the mRNA expression of PPAR-*γ* in NPC is lower than NPG (Figure 2(a)). Western blotting revealed stronger E2F2 expression in nonkeratinizing NPC tissues and weaker PPAR-*γ* expression in nonkeratinizing NPC tissues when compared with NPG tissues (Figure 2(b)), consistent with the IHC findings. A quantitative analysis of the E2F2 and PPAR-*γ* protein expression levels revealed significant differences between the tissue types (P <0.01 and <0.05, respectively) (Figures 2(c) and 2(d)).

### 3.4. PPAR-*γ* Ligand Inhibits the Proliferation of CNE1 and CNE2 Cell Lines

**Figure 3:**
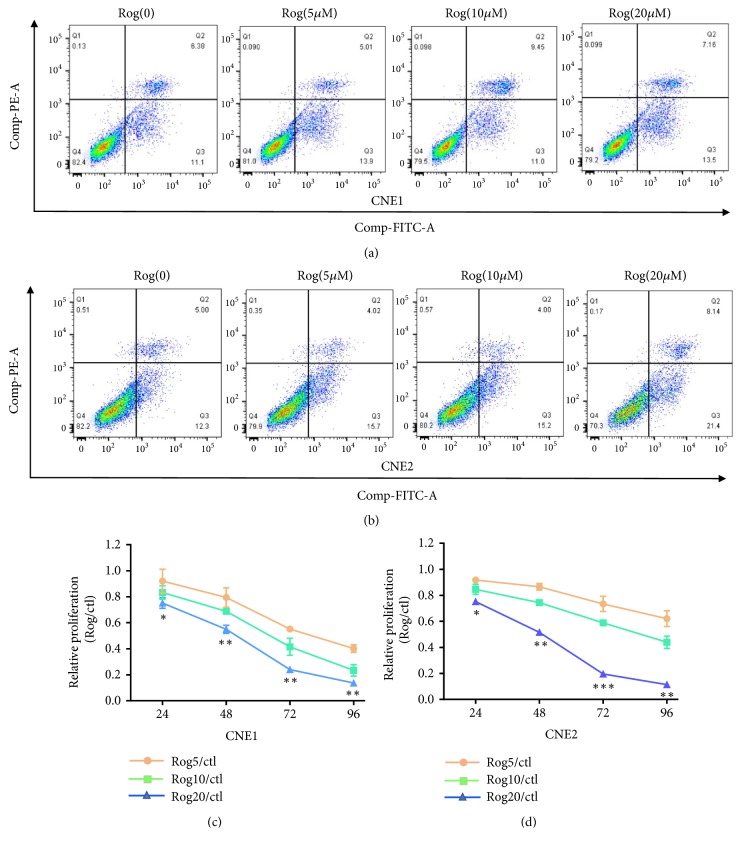
*PPAR-γ ligand inhibits the proliferation of CNE1 and CNE2 cell lines*. (a, b) rh Annexin V-FITC assay was performed to detect the percentage of apoptotic cells relative to the total number of cells in CNE1 and CNE2 cell lines after treatment with dose-dependent Rog. (c, d) Analysis of a CCK-8 proliferation assay of CNE1 and CNE2 cell lines.

To analyze the functional role of PPAR-*γ* in NPC cell lines, we performed CCK-8 assays to detect the proliferation of NPC cell lines. The results showed that PPAR-*γ* ligand, Rog, inhibited the proliferation of both cell lines in a dose-dependent manner (Figures 3(c) and 2(d)). To determine if proliferation was blocked, we performed apoptosis analysis by flow cytometry. The results showed that the number of apoptotic cells did not increase after treatment with Rog (Figures 3(a) and 3(b)).

### 3.5. Expression of E2F2 and PPAR-*γ* in CNE1 and CNE2 Cell Lines after Treatment with a PPAR-*γ* Ligand

**Figure 4:**
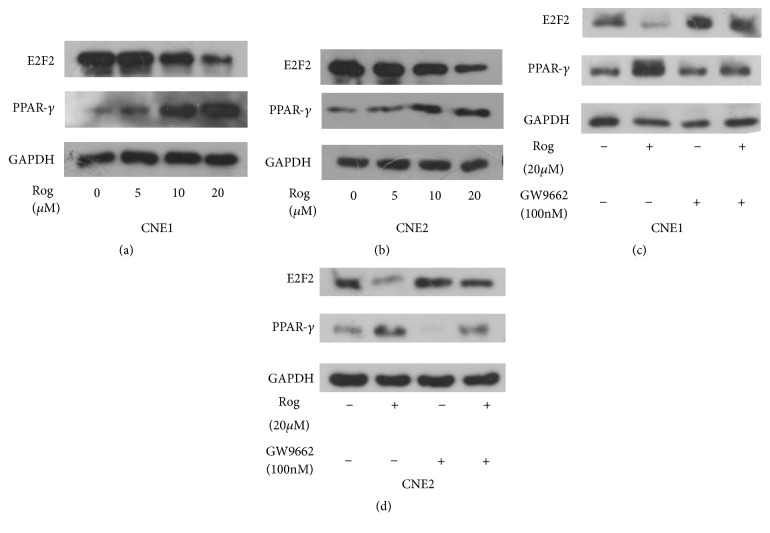
*E2F2 and PPAR-γ expression in CNE1 and CNE2 nasopharyngeal carcinoma cells after treatment with PPAR-γ ligand*. (a, b) Western blotting was performed to detect the expression of E2F2, PPAR-*γ*, and GAPDH in CNE1 and CNE2 cells after treatment with dose-dependent PPAR-*γ* agonist Rog. (c, d) Western blotting was performed to detect the expression of E2F2, PPAR-*γ*, and GAPDH in CNE1 and CNE2 cells after treatment with PPAR-*γ* agonist (rosiglitazone, Rog) and PPAR-*γ* antagonist (GW9662).

Finally, we incubated cell lines with a PPAR-*γ* ligand to analyze the functional role of PPAR-*γ* in NPC cell lines. The expression of E2F2 decreased while PPAR-*γ* increased after treatment with Rog and the effect was dose-dependent (Figures 4(a) and 4(b)). We further verified the protein expression levels using western blotting and found that after treatment with the PPAR-*γ* agonist Rog, the expression of E2F2 decreased whereas that of PPAR-*γ* increased. To confirm the inhibitory effect of PPAR-*γ* on E2F2, we also treated cells with the PPAR-*γ* antagonist GW9662 and observed an increase in the expression of E2F2 (Figures 4(c) and 4(d)). These results suggest that PPAR-*γ* contributes to the inhibition of NPC cell proliferation and metastasis.

## 4. Discussion

NPC is a polygenic, hereditary malignant tumor that most commonly arises from the lateral wall of the nasopharynx, particularly the fossa of Rosenmuller and around the Eustachian cushion (and occult locations). As the primary tumor location is the base of the skull, the local invasion of NPC may have serious consequences, and most patients present with lymph nodes or distant tissue metastasis at the time of diagnosis [15]. Currently, the proliferation and metastasis of tumor tissues are closely related to the differentiation and proliferation rates of cells, understanding that the factors that regulate the cell cycle help us to understand the mechanisms that cause cell proliferation and differentiation, as well as look for factors related to treatment and prognosis.

Previously, we identified that E2F2 could induce the transcription of target genes and drive cell cycle progression and proliferation [16]. Cell cycle deregulation is a common feature of human cancer. The function of proproliferation which lies in the ability enables cell cycle entry into the S phase via E2F2 [17], and strong E2F2 expression was detected in various types of tumors [18–21]. There is no report about E2F2 in NPC. In this study, we use IHC method to analysis 52 nonkeratinizing NPC tissues and 34 NPG tissues revealed stronger E2F2 expression in the former, which was associated with the clinical tumor stage. Specifically, we observed significantly higher levels of E2F2 expression in N2–N3 and M1 stage nonkeratinizing NPC tissues. This finding suggests an association of E2F2 expression with the degree of malignancy, which was confirmed using western blotting and RT-PCR. This finding indicates that E2F2 is a meaningful marker of the degree of tumor malignancy. Furthermore, we found that E2F2 is localized mainly in the nuclei of nonkeratinizing NPC tissues, suggesting that this transcription factor translocates to the nucleus to promote DNA synthesis and subsequent cell proliferation and division. This important role of E2F2 in cell proliferation suggests that treatment with an E2F2 inhibitor may inhibit proliferation.

PPAR-*γ*, a member of the ligand-activated superfamily of nuclear transcription factors, is an important regulator of inflammation, glucose metabolism, and cell proliferation [22] and was previously shown to inhibit tumor cell proliferation [23–25]. In this study, we used IHC, western blotting and RT-PCR to demonstrate reduced PPAR-*γ* expression in nonkeratinizing NPC tissues compared to NPG tissues, especially in cases of the former that involved distant metastasis. It suggests that expression of PPAR-*γ* related to NPC and related to the malignancy degree of tumor. The antitumor affections of PPAR-*γ* were thought to be associated with inhibition of angiogenesis, stemness process, and cell cycle arrest [26, 27]. In our study, we observed a decrease in E2F2 expression and reductions in the proliferation and differentiation rates in NPC cells treated with the PPAR-*γ* agonist Rog and it was dose-dependent. Notably, this effect was offset by incubation with the PPAR-*γ* agonist GW9662. Supporting our result, Komatsu et al. reported that the downregulation of E2F2 led to PPAR-*γ* agonist-mediated cell cycle withdrawal, leading to a cell cycle blockade at the G1/S transition. Numerous studies had demonstrated that ligand activation of PPAR-*γ* activation inhibits the phosphorylation of Rb protein, which has been reported to lead to abrogation of E2F2 [28] or directly decrease the DNA-binding activity of E2F/DP transcription factors [29]. Wu et al. found that combined inactivation of E2F2 is sufficient to block cellular proliferation completely [30].

Potentially, PPAR-*γ* partly downregulates the expression of E2F2 and thus slows the proliferation of tumor cells. Thus, PPAR-*γ* may be a new therapeutic target for NPC.

## Figures and Tables

**Table 1:** Expressions of E2F2 and PPAR-*γ* in clinical samples of undifferentiated NPC and NPG and associations with clinicopathological characteristics of undifferentiated NPC samples.

Parameter	N	E2F2^a^			N	PPAR-*γ*^b^		
		Low expression	High expression	P value^c^		Low expression	High expression	P value^c^
Histologic type				<0.01				<0.01
NPC	52	19 (36.54)	33 (63.46)		52	37 (71.15)	15 (28.85)	
NPG	34	28 (82.35)	6 (17.65)		34	8 (23.53)	26 (76.47)	
Gender				0.868				0.377
Male	39	14 (35.90)	25 (64.10)		39	29 (74.36)	10 (25.64)	
Female	13	5 (38.46)	8 (61.54)		13	8 (61.54)	5 (38.46)	
Age (y)				0.253				0.1
<50	22	10 (45.45)	12 (54.55)		22	13 (59.09)	9 (40.91)	
≧50	30	9 (30.00)	21 (70.00)		30	24 (80.00)	6 (20.00)	
T classification				0.457				0.33
I-II	35	14 (40.00)	21 (60.00)		35	23 (65.71)	12 (34.29)	
III-IV	17	5 (29.41)	12 (70.59)		17	14 (82.35)	3 (17.65	
N classification				0.037				0.071
N0-N1	40	18 (45.00)	22 (55.00)		40	26 (65.00)	14 (35.00)	
N2-N3	12	1 (8.33)	11 (91.67)		12	11 (91.67)	1 (8.33)	
M classification				0.004				0.046
M0	42	19 (45.24)	23 (54.76)		42	27 (64.29)	15 (35.71)	
M1	10	0 (0)	10 (100)		10	10 (100)	0 (0)	

^a^E2F2 low expression: IOD/Area<0.02; high expression: IOD/Area*⩾*0.02.

^b^PPAR-*γ* low expression: IOD/Area<0.02; high expression: IOD/Area*⩾*0.02.

^c^Chi-square test.

## Data Availability

The data used to support the findings of this study are available from the corresponding author upon request.

## Abbreviations

PPAR-*γ*: Peroxisome proliferator-activated receptor-*γ*
E2F2: E2F transcription factor 2
NPC: Nasopharyngeal carcinoma
NPG: Nasopharyngitis
Rog: Rosiglitazone.
